# Supplementary material for: Clonal associations between lymphocyte subsets and functional states in rheumatoid arthritis synovium
Source: Nat Commun. 2024 Jun 11;15:4991. doi: 10.1038/s41467-024-49186-0 (PMC11167034; doi:10.1038/s41467-024-49186-0)
Supplement: Supplementary file 1 — Supplementary Information [file 41467_2024_49186_MOESM1_ESM.pdf]

Supplementary Information

Clonal associations between lymphocyte subsets and functional states in rheumatoid arthritis synovium

Garrett Dunlap[1,*], Aaron Wagner[2,*], Nida Meednu[3,*], Ruoqiao Wang[4], Fan Zhang[1,5,6,7,8,9], Jabea Cyril Ekabe[3], A. Helena Jonsson[1], Kevin Wei[1], Saori Sakaue[1,5,6,7,8], Aparna Nathan[1,5,6,7,8], Accelerating Medicines Partnership Program: Rheumatoid Arthritis and Systemic Lupus Erythematosus (AMP RA/SLE) Network, Vivian P. Bykerk[10,11], Laura T. Donlin[10,11], Susan M. Goodman[10,11], Gary S. Firestein[12], David L. Boyle[12], V. Michael Holers[13], Larry W. Moreland[13,14], Darren Tabechian[3], Costantino Pitzalis[15,16,17], Andrew Filer[18,19,20], Soumya Raychaudhuri[1,5,6,7,8], Michael B. Brenner[1], Juilee Thakar[2,4], Andrew McDavid[2,#], Deepak A. Rao[1,#,&], Jennifer H. Anolik[3,4,#,&]

Affiliations:
[1]Division of Rheumatology, Inflammation, and Immunity, Department of Medicine, Brigham and Women's Hospital and Harvard Medical School; Boston, MA, USA
[2]Department of Biostatistics and Computational Biology, University of Rochester School of Medicine and Dentistry; Rochester, NY, USA
[3]Division of Allergy, Immunology and Rheumatology, University of Rochester Medical Center; Rochester, NY, USA
[4]Department of Microbiology and Immunology, University of Rochester School of Medicine and Dentistry; Rochester, NY, USA
[5]Center for Data Sciences, Brigham and Women's Hospital; Boston, MA, USA
[6]Division of Genetics, Department of Medicine, Brigham and Women's Hospital; Boston, MA, USA
[7]Department of Biomedical Informatics, Harvard Medical School; Boston, MA, USA
[8]Broad Institute of MIT and Harvard; Cambridge, MA, USA
[9]Division of Rheumatology and the Center for Health Artificial Intelligence, University of Colorado School of Medicine; Aurora, CO, USA
[10]Hospital for Special Surgery; New York, NY, USA
[11]Weill Cornell Medicine; New York, NY, USA
[12]Division of Rheumatology, Allergy and Immunology, University of California, San Diego; La Jolla, CA, USA
[13]Division of Rheumatology, University of Colorado School of Medicine; Aurora, CO, USA
[14]Division of Rheumatology and Clinical Immunology, University of Pittsburgh School of Medicine; Pittsburgh, PA, USA
[15]Centre for Experimental Medicine & Rheumatology, EULAR Centre of Excellence, William Harvey Research Institute, Queen Mary University of London; London, UK
[16]Barts Health NHS Trust, Barts Biomedical Research Centre (BRC), National Institute for Health and Care Research (NIHR), London, UK
[17]Department of Biomedical Sciences, Humanitas University and Humanitas Research Hospital, Milan, Italy
[18]Rheumatology Research Group, Institute for Inflammation and Ageing, University of Birmingham, NIHR Birmingham Biomedical Research Center and Clinical Research Facility, University of Birmingham, Queen Elizabeth Hospital; Birmingham, UK
[19]Birmingham Tissue Analytics, Institute of Translational Medicine, University of Birmingham, Birmingham, UK
[20]NIHR Birmingham Biomedical Research Center and Clinical Research Facility, University of Birmingham, Queen Elizabeth Hospital, Birmingham, UK.
[*]These authors contributed equally: Garrett Dunlap, Aaron Wagner, Nida Meednu
[#]These authors jointly supervised this work: Andrew McDavid, Deepak A. Rao, Jennifer H. Anolik
[%]Corresponding authors

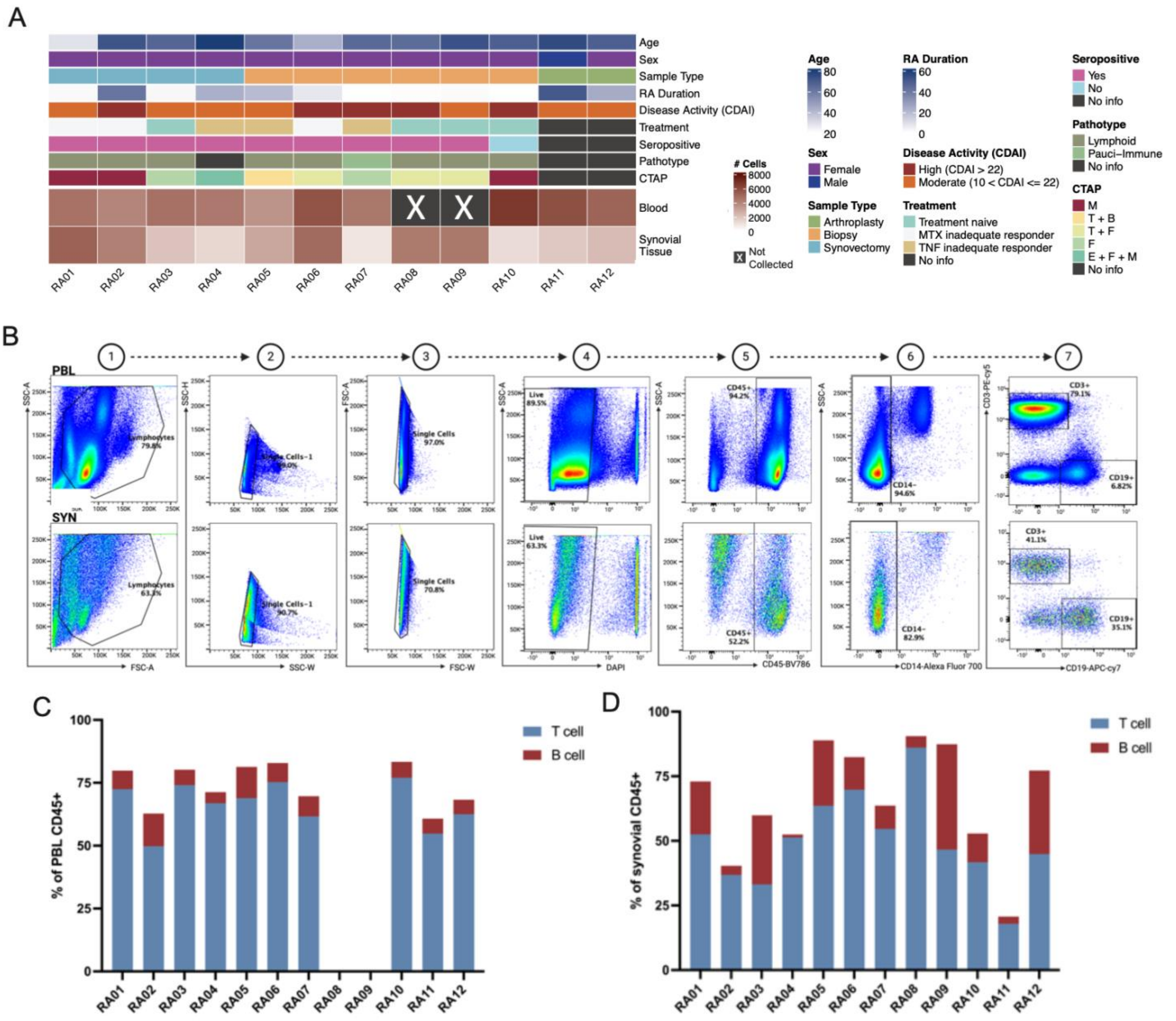

Supplementary Fig. 1 A. Heat map highlighting the cell recovery for each patient, along with metadata associated with the cohort. Cell Type Abundance Phenotype (CTAP) was defined as in Zhang et al. 2023. **B.** Stepwise flow sorting scheme for blood and synovial tissue samples. **C.** Percent of sorted T and B cells among CD45+ cells in peripheral blood lymphocytes. **D.** Percent of sorted T and B cells among CD45+ cells in synovial tissue.

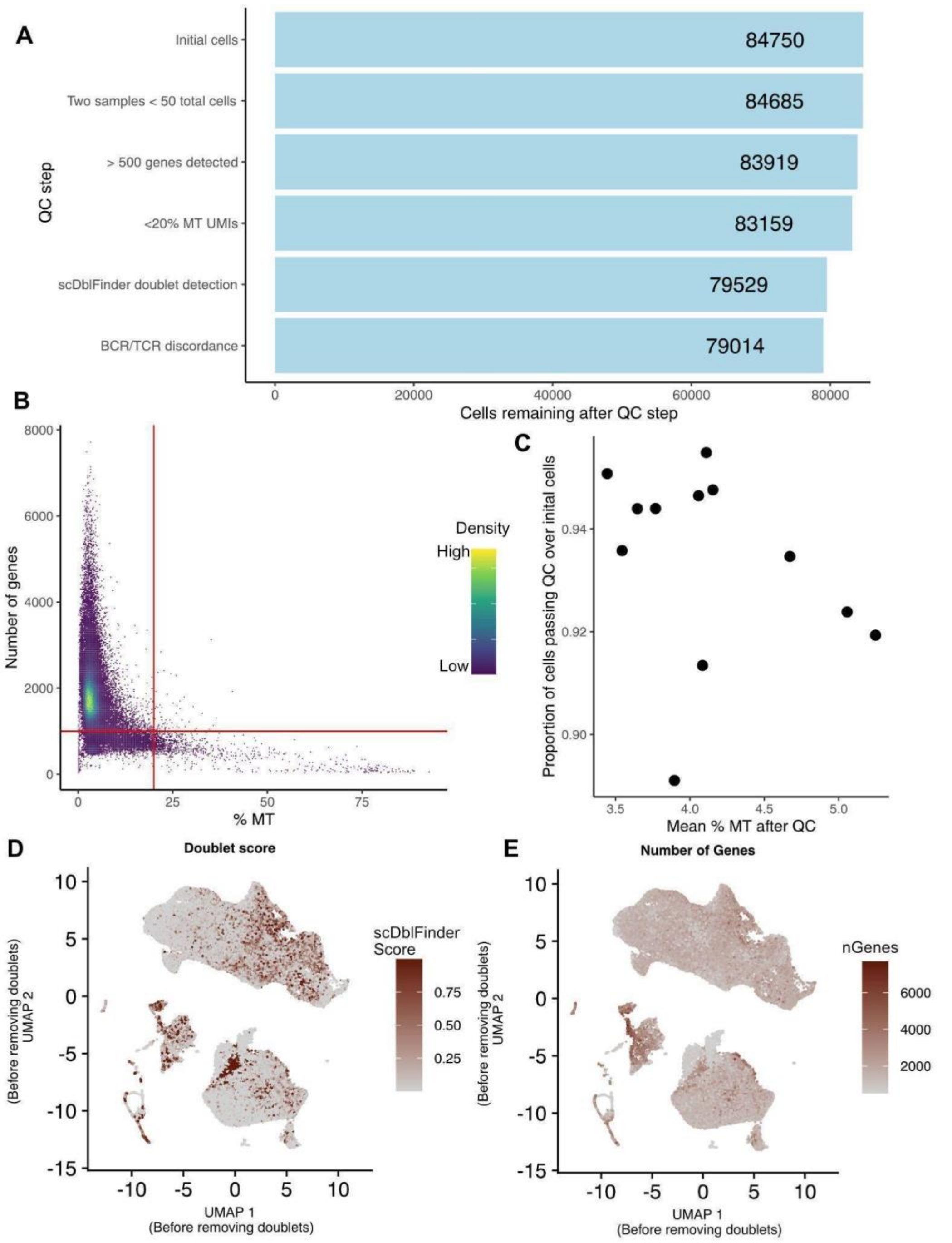

Supplementary Fig. 2 A. Bar plot of initial quality control filters showing the number of cells remaining after each quality control step. **B.** Scatter plot of the number of detected genes and the percent of mitochondrial reads for each cell. Red lines correspond to the selected QC filters (>500 genes and <20% MT reads). **C.** Scatter plot of mean mitochondrial percentage and proportion of cells passing QC for each sample. Each dot corresponds to a single sample (n=12 samples). **D.** UMAP showing doublet scores according to scDblFinder of all cells before QC. Color scale represents a doublet score for each cell. **E.** UMAP showing the number of genes in each cell before QC. Color scale defines number of genes per cell before QC.

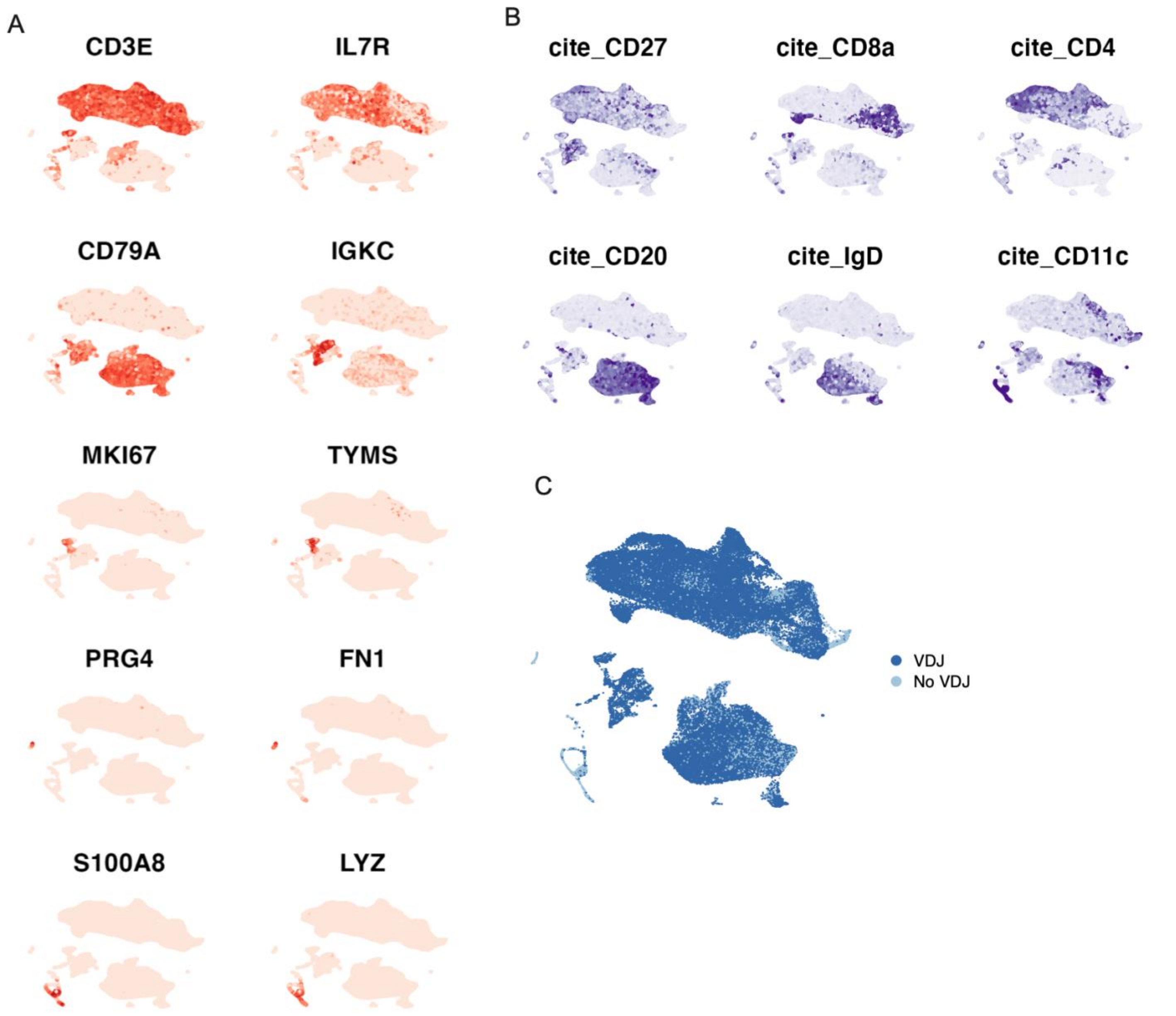

Supplementary Fig. 3 A. UMAPs of the gene expression levels for select markers used to identify each population. **B.** UMAPs of the detection of CITEseq barcoded antibodies used in this study. The darker the color the higher the expression of that gene (A) or protein (B). **C.** UMAP of the distribution of cells that have associated VDJ information.

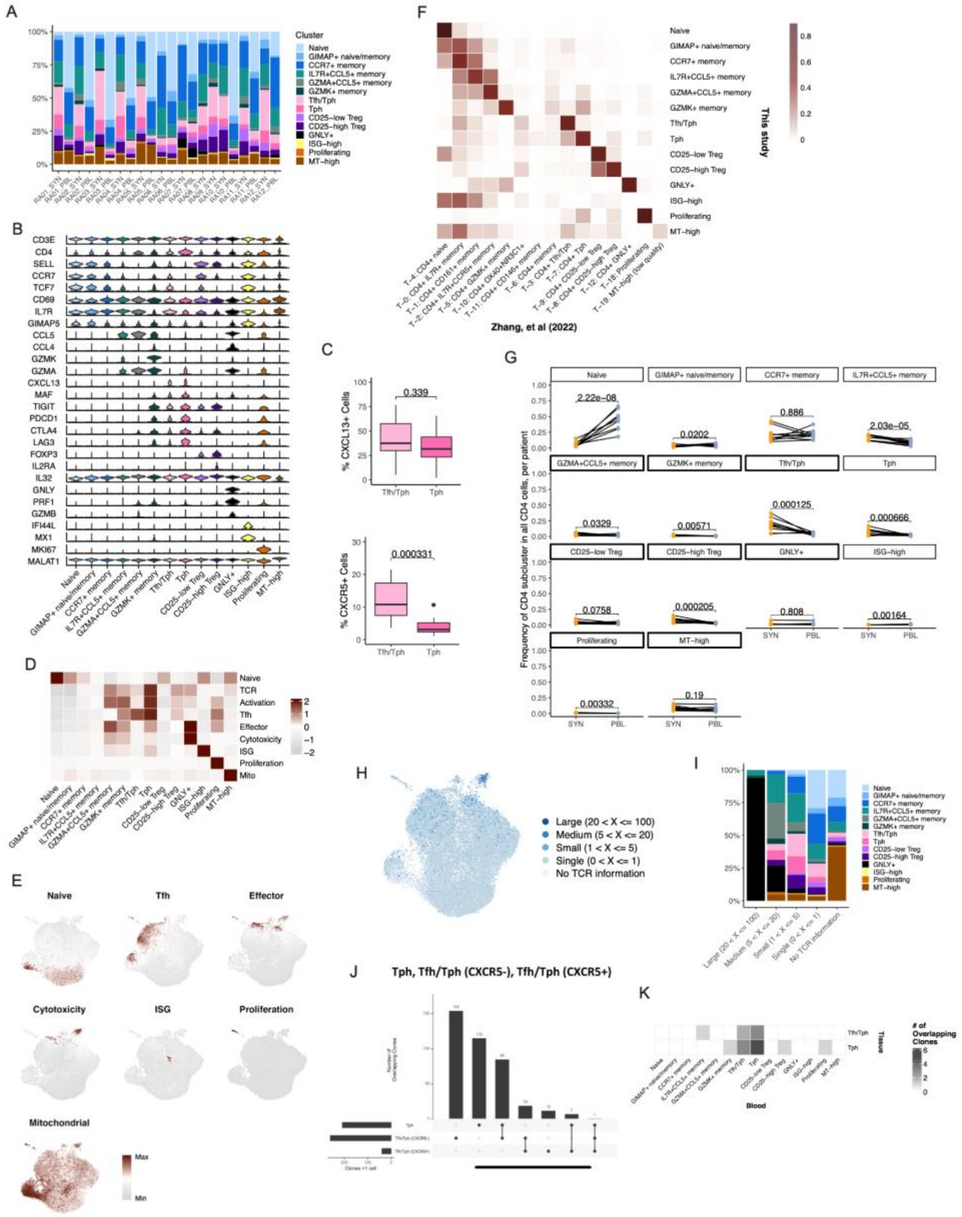

Supplementary Fig. 4 A. Bar plot of the CD4+ cluster composition for each sample. **B.** Violin plots of the expression distribution of select, differentially-expressed markers for each cluster. **C.** Box plots of the percent of cells per patient expressing *CXCL13* and *CXCR5* in the Tfh/Tph and Tph clusters (n=12 donors). **D.** Heat map showing the scaled module score of select gene signatures. **E.** UMAPs highlighting the enrichment of select gene signatures. **F.** Heat map of the confidence for each cluster to match the CD4+ T cell clusters of a previously-described single-cell synovial tissue dataset. **G.** For each cluster, frequency of cell representation as a proportion of all CD4 cells in a sample. Each dot represents a single sample, and lines denote paired blood and tissue for a donor (n=12 donors). Significance is determined by paired T-tests, with multiple testing correction. **H.** UMAP of the clonal expansion among CD4+ cells. **I.** Bar plot of the distribution of clusters across each clone size category. **J**. UpSet plot of shared clones between Tph cluster and cells from Tfh/Tph cluster divided based on *CXCR5* transcript detection. **K.** Heatmap of the numbers of Tph of Tph/Tfh synovial clones shared across clusters in the blood. For C, box plots are defined with lower and upper edges corresponding to the first and third quartiles (the 25th and 75th percentiles), respectively, the upper whisker extends from the 75th percentile to the largest value no further than 1.5x the interquartile range (IQR) from the edge, the lower whisker extends from the edge to the smallest value at most 1.5x the IQR of the lower edge, and the median is the center line.

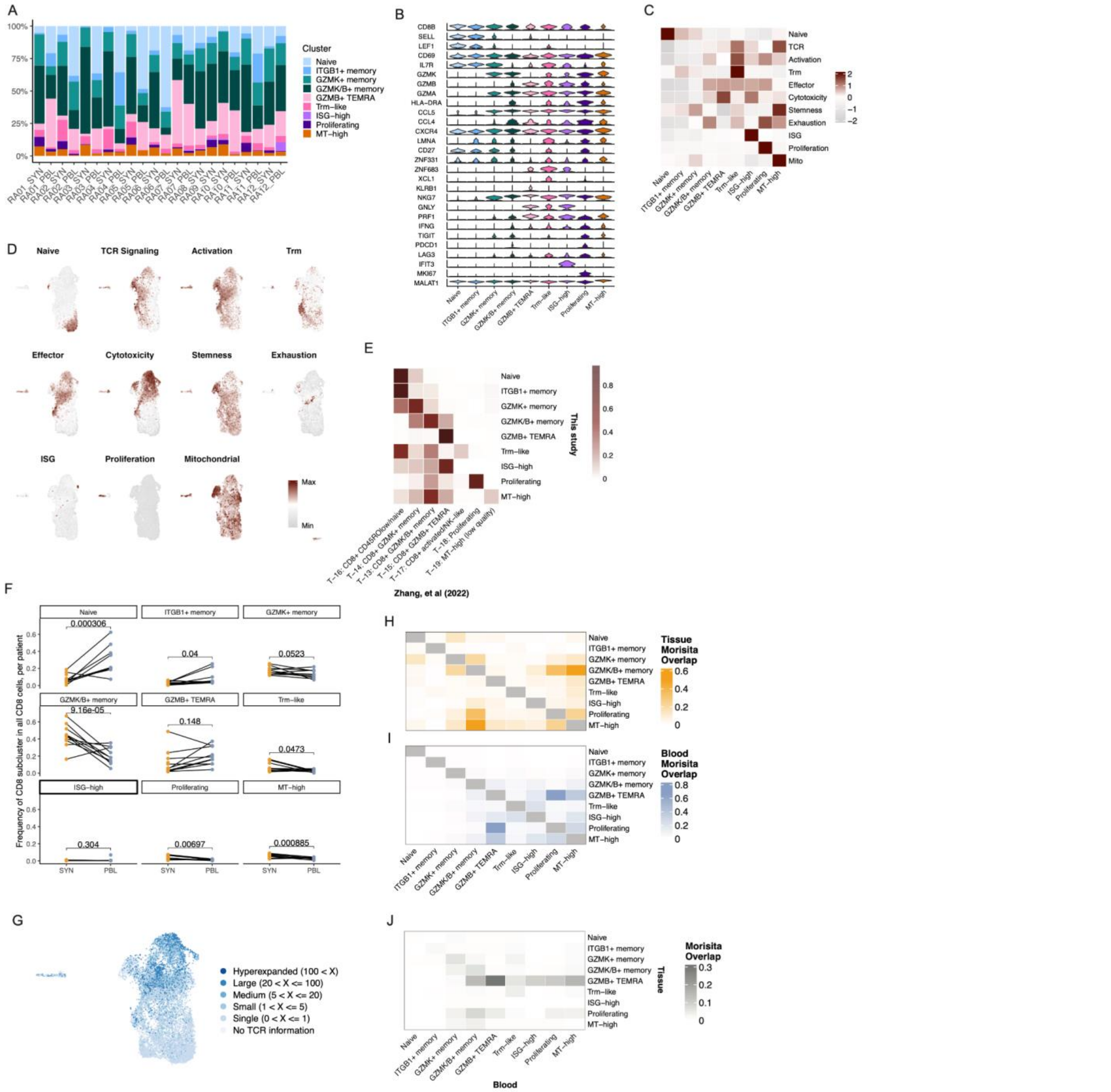

Supplementary Fig 5. A. Bar plot of the CD8+ cluster composition for each sample. **B.** Violin plots of the expression distribution of select, differentially-expressed markers for each cluster. **C.** Heat map showing the scaled module score of select gene signatures. **D.** UMAPs highlighting the enrichment of select gene signatures. **E.** Heat map of the confidence for each cluster to match the CD8+ T cell clusters of a previously-described single-cell synovial tissue dataset. **F.** For each cluster, frequency of cell representation as a proportion of all CD4 cells in a sample. Each dot represents a single sample, and lines denote paired blood and tissue for a donor (n=12 donors). Significance is determined by paired T-tests, with multiple testing correction. **G.** UMAP of the clonal expansion among CD8+ cells. **H and I**. Heat map of pairwise clonal overlap values calculated using Morisita's index for synovial tissue **(H)** and blood **(I)**. **J.** Heat map of pairwise clonal overlap values between tissue and blood calculated using Morisita's index.

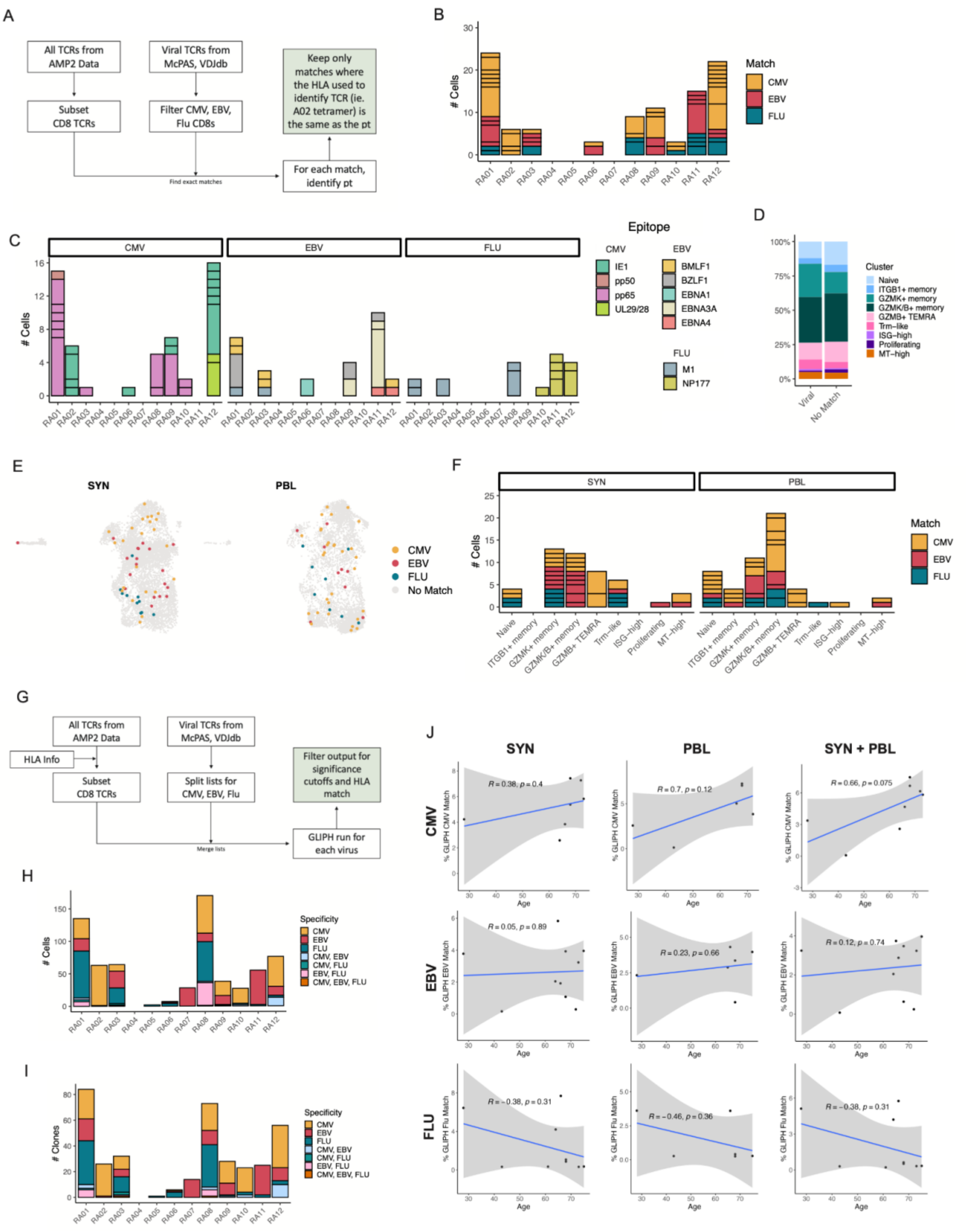

Supplementary Fig 6. A. Schematic diagram of exact TCR matching with viral-specific databases. **B.** Bar plot of the number of exact viral-reactive matching cells per patient, split by virus. Box size denotes size of clone. **C.** Bar plot of exact matches per patient, split by virus and epitope. **D.** Bar plot of the cluster distribution for exact viral matching and non-matching cells. **E.** UMAP of exact viral reactive matching cells, split by virus and tissue. **F.** Bar plot of exact matches per cluster, split by virus and tissue. **G.** Schematic diagram of TCR motif finding with viral-specific databases using GLIPH2. **H.** Bar plot of the number of GLIPH2 motif viral-reactive matching cells per patient, split by virus. **I.** Bar plot of unique clones with virus match using GLIPH2 per patient, split by virus. **J.** Scatter plots of age and percent virus matching CD8+ T cells using GLIPH2, split by tissue. PBL, peripheral blood lymphocytes. SYN, synovial tissue.

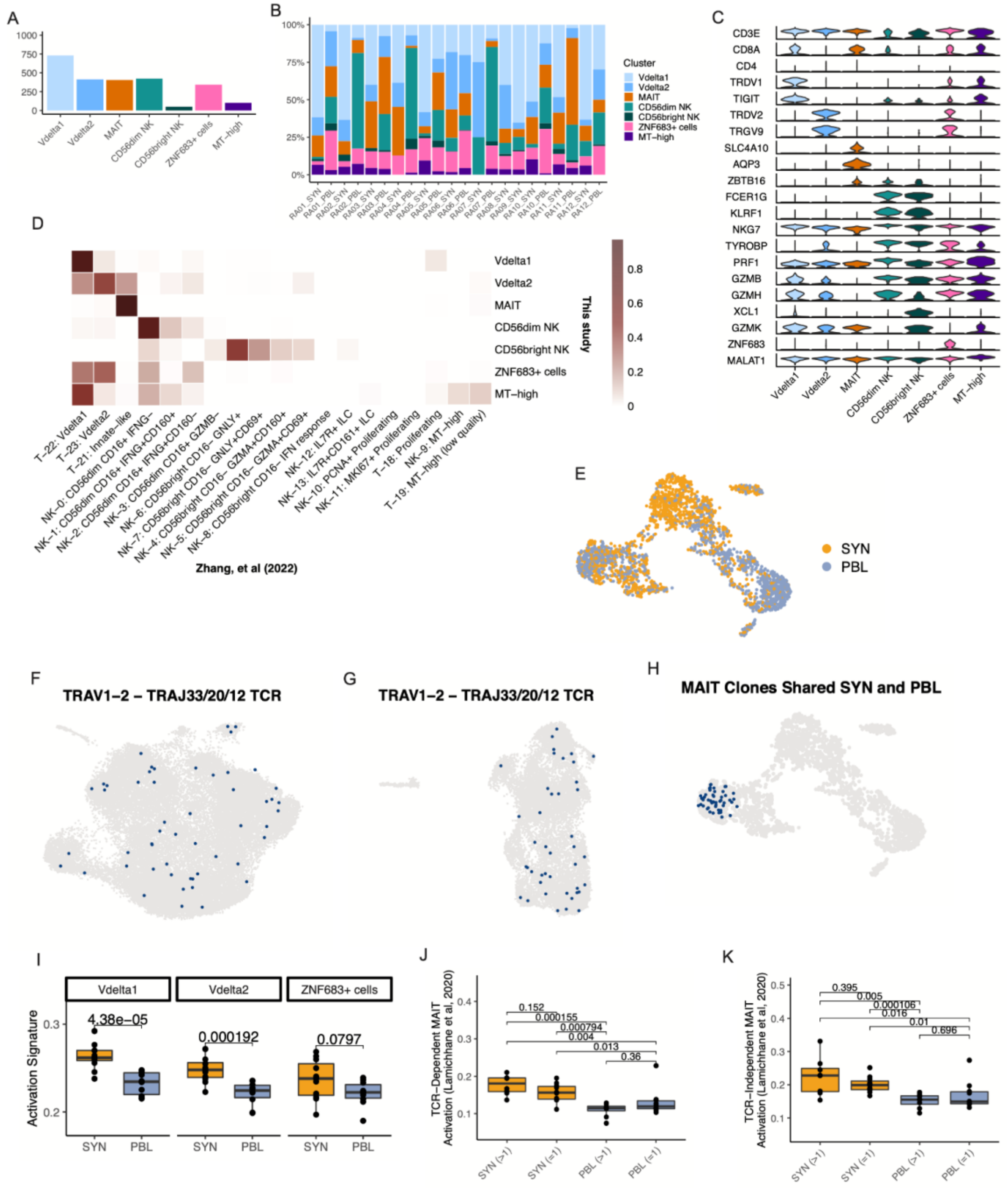

Supplementary Fig 7. A. Bar plot of the number of cells included in each cluster. **B.** Bar plot of the CD4+ cluster composition for each sample (n=12 samples). **C.** Violin plots of the expression distribution of select, differentially-expressed markers for each cluster. **D.** Heat map of the confidence for each cluster to match the innate T and NK cell clusters of a previously-described single-cell synovial tissue dataset. **E.** UMAP showing the distribution of tissue source. UMAPs highlighting CD4+ **(F)** and CD8+ **(G)** T cells with a TRAV1-2 and either TRAJ33, 20, or 12 gene rearrangement. **H.** UMAP highlighting MAIT clones found in both blood and synovial tissue of a donor. **I.** Box plot of activation signature across innate T cell clusters. **J and K.** TCR-dependent **(J)** and independent **(K)** MAIT cell activation signatures from Lamichhane et al, 2020, split by tissue and clonal expansion. Significance was calculated using paired Wilcox testing with Holm correction. For I-K, box plots are defined with lower and upper edges corresponding to the first and third quartiles (the 25th and 75th percentiles), respectively, the upper whisker extends from the 75th percentile to the largest value no further than 1.5x the interquartile range (IQR) from the edge, the lower whisker extends from the edge to the smallest value at most 1.5x the IQR of the lower edge, and the median is the center line.

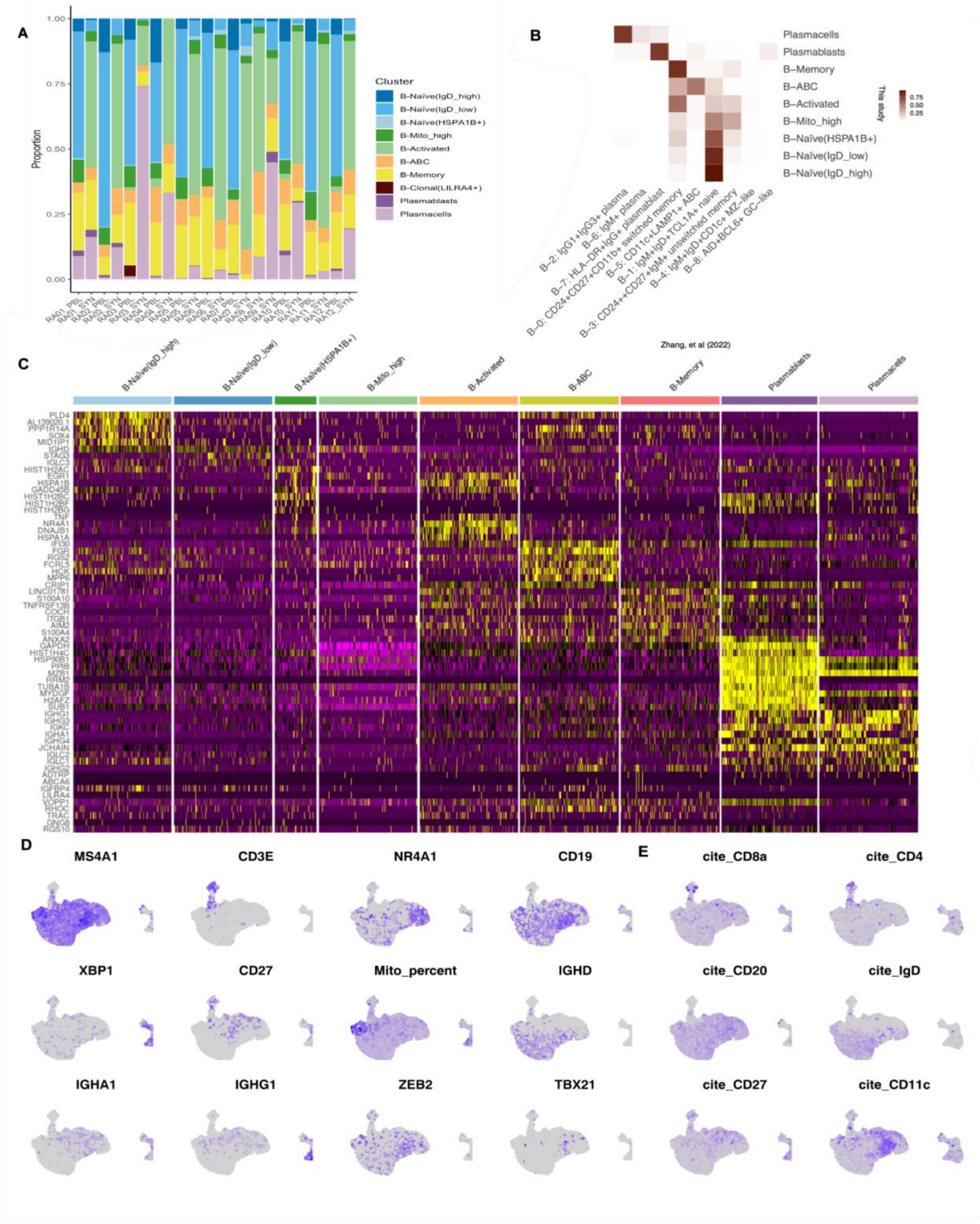

Supplementary Fig. 8 A. Bar plot of the B cell cluster composition for each sample. **B.** Heat map of the confidence for each cluster to match the B cell clusters of a previously described single-cell synovial tissue dataset. **C.** Heatmap of top 10 differentially expressed genes for each cell population according to average log2-fold change. **D.** UMAP of additional markers used to identify cell types. **E.** UMAP of cite seq markers available in this dataset.

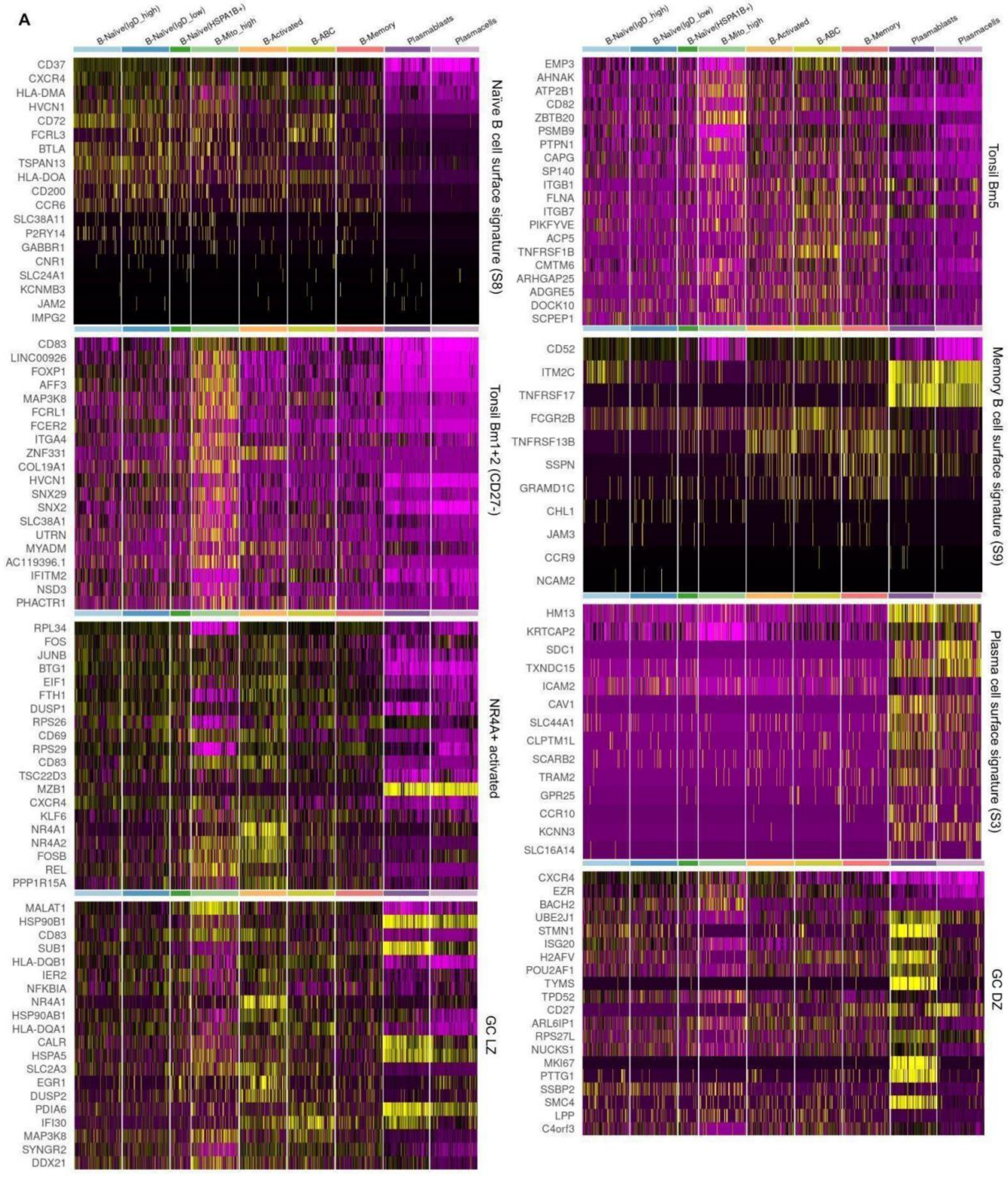

Supplementary Fig. 9 A. Heatmaps of 8 selected gene signatures from Figure 5E.

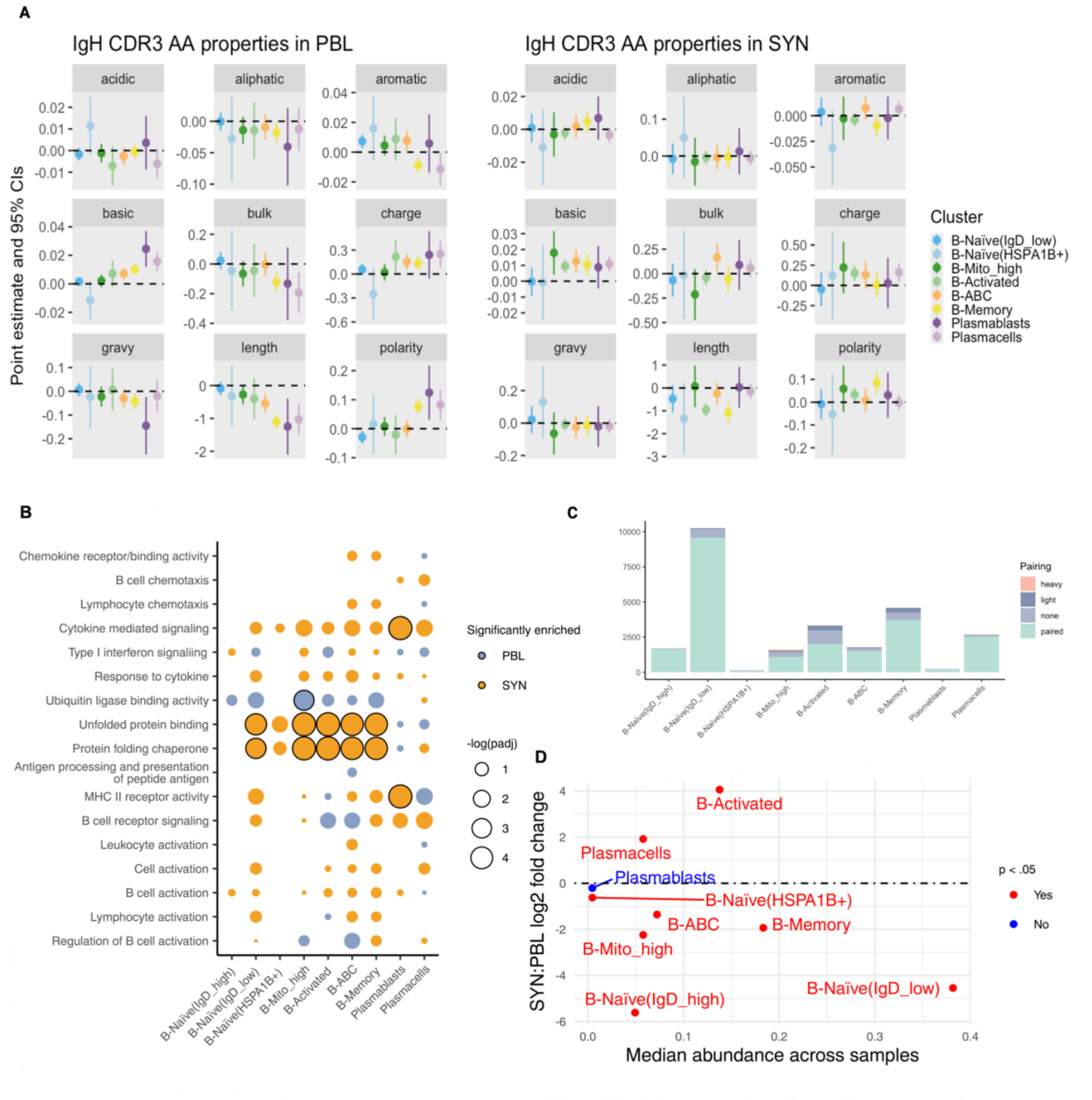

Supplementary Fig. 10 A. Plot showing the effect size and 95% confidence intervals of population on IgH CDR3 amino acid properties using all B-Naive(IgD-high) cells (SYN and PBL) as the reference population, split by tissue. Center of the error bar is the random intercept estimated by a linear mixed-effects model using amino acid properties as the dependent variable with a fixed effect for clusters. **B.** Dotplot of selected pathways checked for gene set enrichment between blood and synovium cells for each population. Dots outlined in black deemed significantly enriched. p.adjust < 0.05. C. Bar chart of Immunoglobulin chain pairings for each cell population. **D.** Plot showing the median sample abundance of each cluster and the synovium to peripheral blood log2 fold change for each cluster, significance determined according to mixed-effect model specified using Mixed-effects modeling of Associations of Single Cells (MASC). Dots above the dash line indicate populations more abundant in synovium (SYN) over blood (PBL) whereas dots below the dash line represent populations more abundant in blood. Red dots label significant abundance (p < 0.05) and blue dots are not significant.

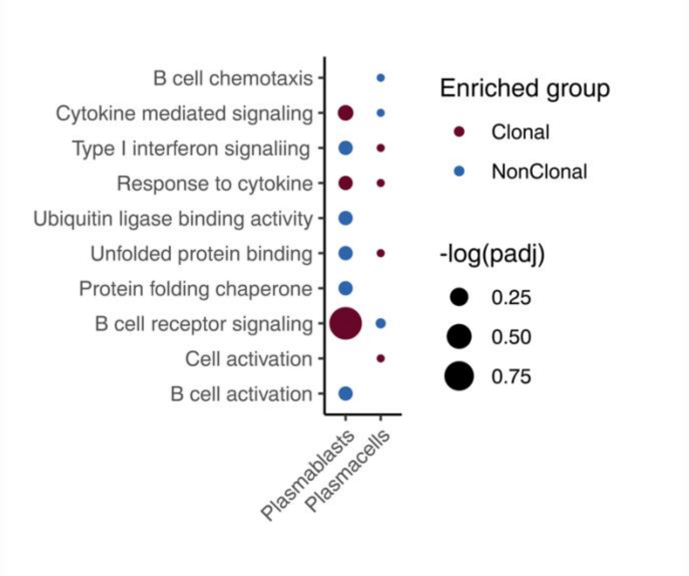

Supplementary Fig. 11 Dot plot of selected pathways checked for gene set enrichment between clonal cells (red) and non-clonal cells (blue) in the synovium. Cell states were omitted that had a limited number of clones. The size of the circle represents -log(p-adjust). Multiple testing correction is performed to get adjusted p-values.

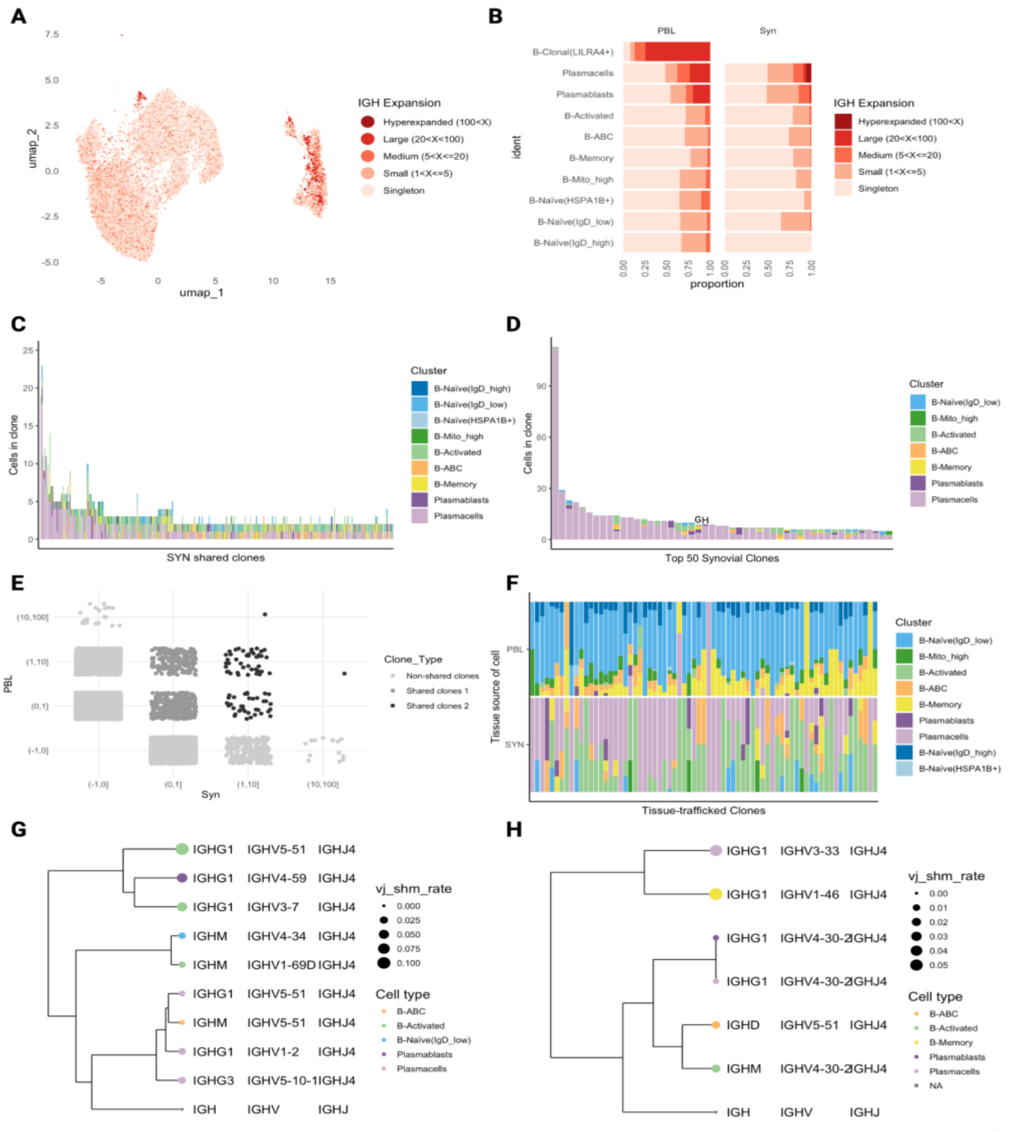

Supplementary Fig 12. Clones are defined using a similarity threshold of 80%. A. UMAP projection of IgH-defined clonal expansion in B cells. Cells without BCR are not included in this plot or other plots in this figure. **B.** Bar chart showing the proportion of clone sizes for each cluster split by synovium (Syn) and blood (PBL). **C.** Bar plot showing the cell identity and clone size for each synovial clone shared by two or more B cell states **D.** Bar plot displaying the top 50 largest clones in the synovium and their cell population composition. G and H depict clones with clonal tree examples in panels G and H, respectively. **E.** Plot displaying the distribution of clones across each tissue and clones shared between tissues. **F.** Plot of cell population composition of the tissue-trafficked clones that are expanded (1-10 cells clone size) in the synovium (black dots in **E**). **G**. B cell lineage tree of clone G from panel D. **H.** B cell lineage tree of clone H from panel D. B cell states are depicted by different colors. The size of the circle corresponds to the V-J somatic hypermutation (shm) rate compared to the germline which was calculated by dividing the sum of V-shm and J-shm by the sequence length excluding the CDR3 region. The isotype of each end node is shown. The distance matrix was computed using the Euclidean method and the ward.D method for hierarchical clustering and dendrogram plot construction.

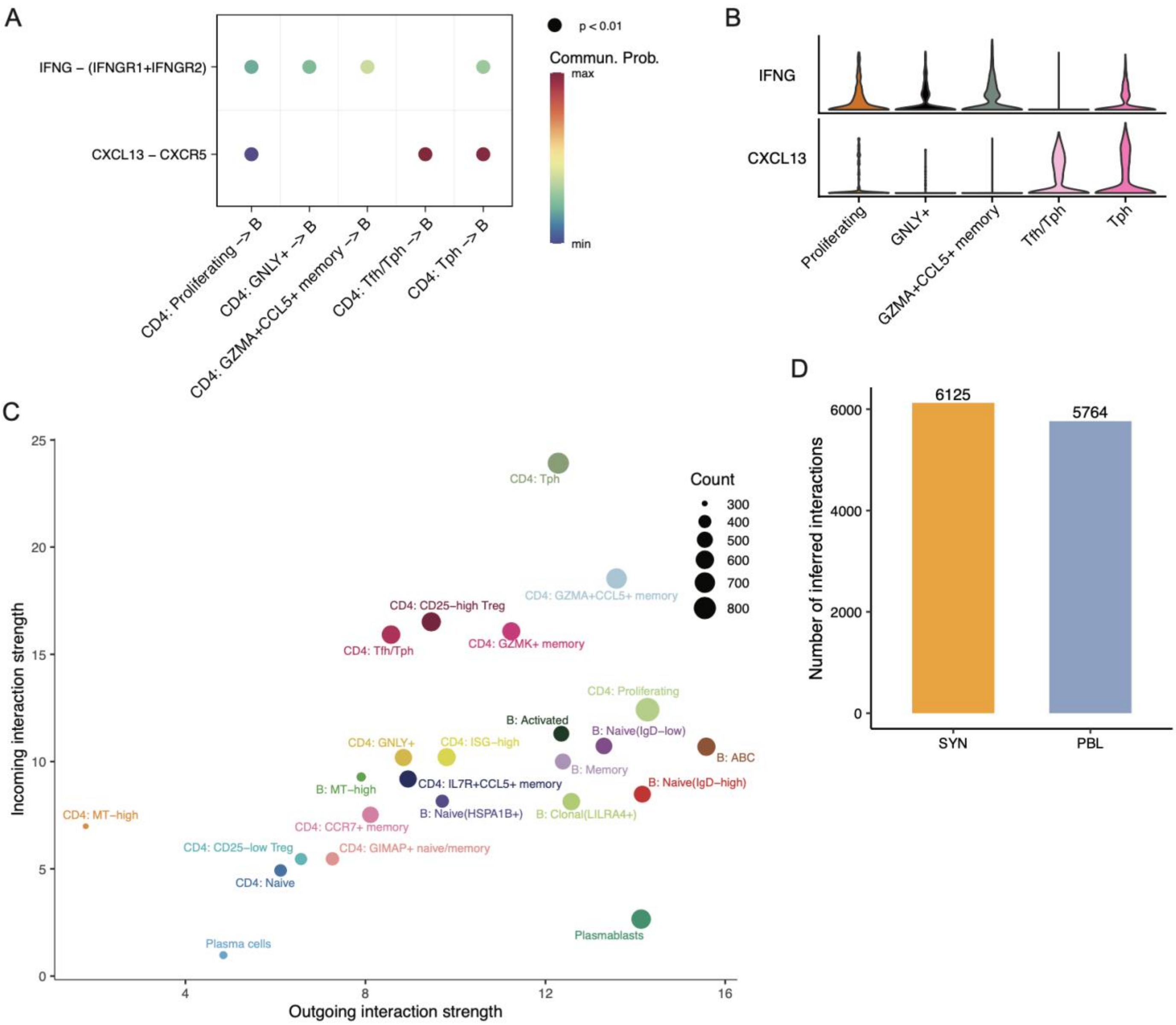

Supplementary Fig. 13 A. Dot plot of select signaling pathways between CD4+ T cell subsets and B cells, with significance assessed by a permutation test (n = 100 permutations). **B.** Violins of the expression of IFNG and CXCL13 in the CD4+ T cell subsets identified in (A). **C.** Scatter plot of the computed incoming and outgoing signaling strengths for each CD4+ T and B cell subpopulation. **D.** The number of significant interactions detected in each tissue. PBL, peripheral blood lymphocytes. SYN, synovial tissue.
