# Supplementary material for: Clonal associations between lymphocyte subsets and functional states in rheumatoid arthritis synovium
Source: Nat Commun. 2024 Jun 11;15:4991. doi: 10.1038/s41467-024-49186-0 (PMC11167034; doi:10.1038/s41467-024-49186-0)
Supplement: Supplementary file 3 — Reporting Summary [file 41467_2024_49186_MOESM3_ESM.pdf]

Corresponding author(s): Deepak A. Rao, Jennifer H. Anolik

Last updated by author(s): Feb 15, 2024

Reporting Summary

Nature Portfolio wishes to improve the reproducibility of the work that we publish. This form provides structure for consistency and transparency in reporting. For further information on Nature Portfolio policies, see our Editorial Policies and the Editorial Policy Checklist.

Statistics

For all statistical analyses, confirm that the following items are present in the figure legend, table legend, main text, or Methods section.

n/a	Confirmed	
☐	☒	The exact sample size (*n*) for each experimental group/condition, given as a discrete number and unit of measurement
☐	☒	A statement on whether measurements were taken from distinct samples or whether the same sample was measured repeatedly
☐	☒	The statistical test(s) used AND whether they are one- or two-sided<br>*Only common tests should be described solely by name; describe more complex techniques in the Methods section.*
☐	☒	A description of all covariates tested
☐	☒	A description of any assumptions or corrections, such as tests of normality and adjustment for multiple comparisons
☐	☒	A full description of the statistical parameters including central tendency (e.g. means) or other basic estimates (e.g. regression coefficient) AND variation (e.g. standard deviation) or associated estimates of uncertainty (e.g. confidence intervals)
☐	☒	For null hypothesis testing, the test statistic (e.g. *F*, *t*, *r*) with confidence intervals, effect sizes, degrees of freedom and *P* value noted<br>*Give P values as exact values whenever suitable.*
☒	☐	For Bayesian analysis, information on the choice of priors and Markov chain Monte Carlo settings
☐	☒	For hierarchical and complex designs, identification of the appropriate level for tests and full reporting of outcomes
☒	☐	Estimates of effect sizes (e.g. Cohen's *d*, Pearson's *r*), indicating how they were calculated

Our web collection on statistics for biologists contains articles on many of the points above.

Software and code

Policy information about availability of computer code

Data collection	Clinical data were collected using REDCap v. 6.9.0 onwards. T cells and B cells were sorted using BD FACSAria II with FACS Diva software V. 8.0.1.<br>Cells collected from cell sorting were encapsulated into oil droplets using a Chromium NextGEM Chip G (10X Genomics).<br>Following reverse transcription and cDNA amplification, 5′ gene expression, immune repertoire, and feature barcode libraries were constructed following manufacturer protocols (v1.1).<br>Gene expression libraries were sequenced to obtain a read depth of 100,000 reads per cell, feature barcode libraries were sequenced at 5,000 reads per cell, and immune repertoire libraries were sequenced at 5,000 reads per cell.<br>FASTQ file demultiplexing for gene expression libraries was performed using the mkfastq function in CellRanger (10X Genomics, v4.0). Following this, alignment to a reference genome (GRCh38) and counting was completed using the count function to generate expression matrices for each sample.<br>Immune repertoire FASTQ files were separately demultiplexed, and the vdj function was used to perform sequence assembly and clonotype calling for TCR and BCR sequences in each sample.
Data analysis	Flow cytometry data were analyzed with FlowJo v. 10.6. Single cell RNA-seq data were aligned and quantified with CellRanger v. 4.0.<br>The following single-cell analyses were performed using R (version ......)<br>Gene count matrices were imported in R for downstream analysis.<br>Quality control was first performed jointly on all cells collected and sequenced in this experiment, including low-quality cell and doublet detection, as well as marking cells that coexpressed at least 1 TCR and 1 BCR.<br>Log-normalization was then applied to the gene expression counts for remaining cells.

Final QC thresholding was performed on the log-normalized counts, keeping cells with greater than 500 genes detected and less than 20 percent of detected reads coming from mitochondrial genes. Additional quality control was performed for T and B cells separately in downstream analysis.

After initial QC, unsupervised clustering was performed on the remaining cells to identify major cell types present in the data. Principal components were first generated in order to reduce the dimensionality of the feature space before clustering.
Using Seurat's clustering functionalities on the first 30 principal components, a 20 nearest-neighbors network graph was computed. Then, we performed Louvain clustering with a resolution parameter of 0.3, and visualized the cells in 2D space using Uniform Manifold Approximation and Projection (UMAP).
Differentially-expressed genes between clusters were identified (Student's T-test) by including only genes exhibiting a greater-than 0.25 log-fold difference between clusters. In order to annotate each cluster with a biologically meaningful name, genes with the highest log-fold changes were considered, as well as marker genes that are cell-type specific.

Cells broadly labeled as "T cells" and "Proliferating" from the combined object were subset, and Harmony was then used to perform batch correction at the level of the patient and tissue using theta = 2 and max.iter.cluster = 20. Using the top 50 Harmony embeddings, Louvain clustering was performed, which was then visualized in UMAP space. Nearest neighbors were identified using the Harmony embeddings, and clustering was iteratively performed with a resolution of 1.4 finally selected. Broad T cell markers (eg. CD4, CD8, TRDC) were used along with differential gene expression (Wilcoxon Rank Sum test) to identify the T cell lineages for each of these initial clusters. At this point, a small number of remaining contaminating cells (such as B cells from the Proliferating cluster), were removed. Based on gene expression, clusters of CD4, CD8, and innate T cells were separated and individually clustered using a similar strategy with slightly different parameters for each subset (CD4: 40 Harmony embeddings, 0.5 cluster resolution; CD8: 40 Harmony embeddings, 0.4 cluster resolution; innate T: 10 Harmony embeddings, 0.4 cluster resolution).

Cells labeled as "B cells" from the broad clustering, we further characterized B cell subpopulations. Before reclustering the B cells, we discard cells marked as doublets according to scDblFinder and B cells which simultaneously coexpressed at least 1 BCR and at least 1 TCR. On the remaining cells, Seurat's default normalization and scaling was performed and principal components were generated. Harmony was used to perform batch correction at the patient level using theta = 2 and max.iter.cluster = 20. From here, the 20-nearest-neighbors network graph was generated using the first 30 harmonized principal components. We then applied Louvain clustering using a resolution parameter of 0.5 to identify clusters of similar cells to visualize in the UMAP space. Differential gene expression was then performed (Student's t-test) to provide markers for cluster annotation. Before selecting the final set of input parameters, results were explored at multiple resolutions, variable number of included principal components, and using both harmonized and non-harmonized principal components.
Script to reproduce analyses are available on GitHub (https://github.com/dunlap/amp2repertoire)

For manuscripts utilizing custom algorithms or software that are central to the research but not yet described in published literature, software must be made available to editors and reviewers. We strongly encourage code deposition in a community repository (e.g. GitHub). See the Nature Portfolio guidelines for submitting code & software for further information.

Data

Policy information about availability of data

All manuscripts must include a data availability statement. This statement should provide the following information, where applicable:

- Accession codes, unique identifiers, or web links for publicly available datasets
- A description of any restrictions on data availability
- For clinical datasets or third party data, please ensure that the statement adheres to our policy

Single cell RNA and TCR/BCR sequencing data generated in this study are available via the ARK portal (doi.org/10.7303/syn47217489.1). he data are available under controlled access due to data privacy laws. To access the data, users need to complete and submit a signed Data Use Certificate (DUC) to the ARK Portal at https://arkportal.synapse.org/Data%20Access. Additional data are provided in the Supplementary Information and as source Data files.

Research involving human participants, their data, or biological material

Policy information about studies with human participants or human data. See also policy information about sex, gender (identity/presentation), and sexual orientation and race, ethnicity and racism.

Reporting on sex and gender	Approximately 92% of participants identified as cisgender women (n=11) and 8% as cisgender men (n=1). Sex and gender were self-reported by the participants. The participants were recruited based on the diagnosis of rheumatoid arthritis (RA) which is a disease that is highly prevalent in female and sex and gender were not considered in the design. Findings of this study do not apply to only one sex or gender
Reporting on race, ethnicity, or other socially relevant groupings	Race and ethnicity were self-reported by the participants at the baseline visit
Population characteristics	The participants were recruited based on the diagnosis of rheumatoid arthritis (RA) exhibiting moderate to high disease activity (100% with CDAI≥10; 80.6% with DAS28-CRP3≥3.2). Participants were recruited across 3 groups: treatment-naive (n=4), methotrexate-inadequate responders (n=3) and anti-TNF agent inadequate responders (n=3). There was no information about treatment group on 2 samples. Participants were between 28-80 years of age, majority are female (n= 11 Female, n= 1 male), n=8 of non-Hispanic and n=4 Hispanic. For race, n=10 are White, n= 1 Asian-White and n=1 black African American. 6 participants reported no smoking history and 4 participants reported yes for smoking history while no information was provided for 2 participants. Some of these covariates are presented in Supplementary Fig 1A.
Recruitment	Synovial biopsies or arthoplasties and matched peripheral blood were collected from patients with RA who were recruited based on the diagnosis of RA and exhibiting moderate to high disease activity (CDAI≥10). Participants were recruited by physician across 13 clinical sites in the United States and 2 sited in the United Kingdom. Participants were recruited mainly at

	academic medical center which may be more likely to see complex cases. Different techniques were used for joint biopsies depend on clinical site and experience of the physician performing the biopsy procedure, which may introduce bias.
Ethics oversight	Samples were collected as part of the Accelerating Medicines Partnership (AMP) Network across 13 clinical sites in the United States and 2 sites in the United Kingdom. The study was performed in accordance with protocols approved by the Institutional Review Board at Standford University (Protocol ID: 33561). All clinical and experimental sites obtained approval for this study from their Institutional Review Boards.

Note that full information on the approval of the study protocol must also be provided in the manuscript.

Field-specific reporting

Please select the one below that is the best fit for your research. If you are not sure, read the appropriate sections before making your selection.

[x] Life sciences [ ] Behavioural & social sciences [ ] Ecological, evolutionary & environmental sciences

For a reference copy of the document with all sections, see nature.com/documents/nr-reporting-summary-flat.pdf

Life sciences study design

All studies must disclose on these points even when the disclosure is negative.

Sample size	No sample size calculation was performed for this study. The samples were a subset of a larger AMP cohort. This study is focused on the repertoire of B cells and T cells, therefore the samples were chosen based on cell yields and clear lymphocyte population by flow cytometry. B cells and T cells from all participants were combined to provide a sufficient number of single B cells and single T cells gene expression and repertoire data.
Data exclusions	Several quality control metrics were applied to the data to exclude low-quality cells from downstream analyses including at least 1000 mapped reads and was not identified as doublets. In addition, cell need to coexpress at least 1 TCR or 1 BCR. Sample from one participant was discarded due to only 12 cells passing these initial QC thresholds. The final QC was performed on the log-normalized counts keeping cells with at least 500 genes and less than 20% mitochondrial genes.
Replication	This study is looking at gene expression and repertoire data at the single cell level. Because each cell is unique and impossible to replicate, there is no possible way to have biological replicates at a single-cell level.
Randomization	Randomization is not relevant to this study. Sequencing data from all cells were integrated with batch correction performed. The same Quality control metrics were performed on all cells.
Blinding	Blinding is not relevant in this study due to the cross-sectional nature of the study. Participants have the same diagnosis and received standard of care treatment. No treatment interventions were provided by the study. Cell clustering were performed regardless of any participant clinical parameters.

Reporting for specific materials, systems and methods

We require information from authors about some types of materials, experimental systems and methods used in many studies. Here, indicate whether each material, system or method listed is relevant to your study. If you are not sure if a list item applies to your research, read the appropriate section before selecting a response.

Materials & experimental systems

n/a	Involved in the study	
[ ]	[x]	Antibodies
[x]	[ ]	Eukaryotic cell lines
[x]	[ ]	Palaeontology and archaeology
[x]	[ ]	Animals and other organisms
[x]	[ ]	Clinical data
[x]	[ ]	Dual use research of concern
[x]	[ ]	Plants

Methods

n/a	Involved in the study	
[x]	[ ]	ChIP-seq
[ ]	[x]	Flow cytometry
[x]	[ ]	MRI-based neuroimaging

Antibodies

Antibodies used	Antibodies used for flow cytometry are listed with clone, dilution, vendor and catalog number (as in Supplementary Data 4): anti-CD19-APC-Cy7 (clone SJ25C1, 1:20, BD biosciences, 557791), anti-CD3-PE-Cy5 (clone UCHT1, 1:5, BD biosciences, 555334), anti-CD14-Alexa Fluor700 (clone M5E2, 1:10, BD biosciences, 557923), anti-CD45 BV786 (clone HI30, 1:20, BD Biosciences, 563716), anti-CD38-PerCP-Cy5.5 (clone HIT2, 1:40, BD Biosciences 551400), anti-CD24-PE-Alexa Fluor610 (clone ML5, 1:40, Invitrogen, MHCD2422).<br>CITE-seq was performed using the following TotalSeq-C antibodies from BioLegend: anti-CD20 - TotalSeq-C (clone 2H7, 1:1, BioLegend, 302363), anti-CD11c-TotalSeq-C (clone S-HCL-3, 1:2, BioLegend, 371521), anti-CD4-TotalSeq-C (clone SK3, 1:5,

	BioLegend, 344651), anti-CD8a-TotalSeq-C (clone RPA-T8, 1:5, BioLegend, 301071), anti-CD27-TotalSeq-C (clone LG.3A10, 1:2, BioLegend, 124245) and anti-IgD-TotalSeq-C (clone IA6-2, 1:1, BioLegend, 348245).
Validation	All flow cytometry antibodies are commercially available and validated for flow cytometry application as stated in the manufacturer's product information.<br>BioLegend TotalSeq-C antibodies are tested by flow cytometry to make sure they stain the expected cell population and that oliogos are attached to the antibodies. The oligomer sequence is confirmed by sequencing. This process has been validated by comparison with a traditional two-step flow cytometry staining as shown on manufacturer website.<br>BioLegend antibodies for flow cytometry: Each lot of antibody is quality control tested by immunofluorescent staining with flow cytometric analysis<br>BD Bioscience antibodies: The specificity of flow cytometry antibodies is confirmed by multiple methodologies including flow cytometry, immunofluorescence, immunohistochemistry or western blot to test staining on a combination of primary cells, cell lines or transfectant models. All antibodies are titrated on the relevant positive or negative cells. Data generated on the relevant primary model at the optimal concentration based on a titration curve can be found in Technical data sheets. Lot-to-Lot variation is controlled by each reagent is bottled to match the previous lot MFI.<br>Thermo Fisher (Invitrogen) anti-CD24 antibody: Each lot of this antibody is tested by flow cytometry using human peripheral blood leukocyte. The testing was performed using 5 ul of antibody per 1x10^6 cells in a 100 ul starting volume.

Flow Cytometry

Plots

Confirm that:

- [x] The axis labels state the marker and fluorochrome used (e.g. CD4-FITC).
- [x] The axis scales are clearly visible. Include numbers along axes only for bottom left plot of group (a 'group' is an analysis of identical markers).
- [x] All plots are contour plots with outliers or pseudocolor plots.
- [x] A numerical value for number of cells or percentage (with statistics) is provided.

Methodology

Sample preparation	For this study, patients were recruited and consented through the Accelerating Medicines Partnership (AMP) Network for RA and SLE (Zhang et al. 2022). Synovial tissue samples and matched peripheral blood mononuclear cells were cryopreserved after collection as described (Donlin et al. 2018). Stored synovial tissue samples were then thawed and disaggregated into single-cell suspensions by mincing and digesting with 100 µg/mL LiberaseTL (Roche) and 100 µg/mL DNaseI (Roche) in RPMI (Life Technologies) for 15 min, with occasional inversion during disaggregation. Disaggregated cells were passed through a 70 µm cell strainer and washed prior to antibody staining with anti-CD235a antibodies (clone 11E4B-7-6 (KC16), Beckman Coulter) and Fixable Viability Dye eFluor 780 (eBioscience/ThermoFisher). Live non-erythrocyte cells (viability dye- CD235-) were collected by fluorescence-activated cell sorting (BD FACSAria Fusion) and were initially cryopreserved in Cryostor CS10 (Sigma-Aldrich). The disaggregated synovial tissue cells and matched cryopreserved peripheral blood mononuclear cells were then thawed in batches, and both T and B cells were collected by fluorescence-activated cell sorting (BD FACSAria II). Maximum of 16,000 of B and T cells combined were sorted from each sample. We sorted 8000 B cells and 8000 T cells for the majority of blood samples. For synovial samples, the number for B cells and T cells sorted varied depending on cell abundance (B cell: 107-7472 cells, T cell: 2616-13636 cells). B cells and T cells from each sample were pooled prior to loading on a Chromium NextGEM Chip G (10X Genomics).
Instrument	BD FACSAria II
Software	FlowJo v10.8 (BD Life Sciences)
Cell population abundance	Mean+/- SD of % CD45+ in the synovium = 80 +/-13.7 (range 55.8-96.4%). Mean+/- SD of % synovial B cells out of CD45+ = 17.7 +/- 13.7 (range 4.19-40.6%). Mean+/- SD of % synovial T cells out of CD45+ = 52.7 +/- 19.6 (range 35.4-70%). Mean+/- SD of % CD45+ in peripheral blood = 93.8 +/-11.3 (range 60-98.8%). Mean+/- SD of % blood B cells out of CD45+ = 7.6+/-2.6 (range 5.59-13.5%). Mean+/- SD of % blood T cells out of CD45+ = 63+/- 9.8 (range 50-76.4%). Cell purity is over 98% determined based on number of T cells and B cells we obtained single cell sequencing data.
Gating strategy	The same gating strategy was used for both synovial tissue and blood. FSC/SSC gate was applied to gate on majority of the cells before removing doublets using step-wise SSC-H/SSC-W then FSC-H/FSC-W. Next, dead cells were excluded. We then used CD45 to gate for hematopoietic cells and exclude monocytes using CD14. B cells were identified as CD19+ and T cells were CD3+.

- [x] Tick this box to confirm that a figure exemplifying the gating strategy is provided in the Supplementary Information.
