# Supplementary material for: Clonal associations between lymphocyte subsets and functional states in rheumatoid arthritis synovium
Source: Nat Commun. 2024 Jun 11;15:4991. doi: 10.1038/s41467-024-49186-0 (PMC11167034; doi:10.1038/s41467-024-49186-0)
Supplement: Supplementary file 4 — Description of Additional Supplementary Files [file 41467_2024_49186_MOESM4_ESM.pdf]

Description of Additional Supplementary Files

Supplementary Data 1: Table summary of patient cohort characteristics (n=12) including demographics, Clinical Disease Activity Index (CDAI), treatment groups (MTX inadequate, TNF inadequate responder, and Treatment naïve), Pathotype, Cell Type Abundance Phenotype (CTAP), medication history, and number of blood and synovial cells after QC. The data are displayed as a heatmap in Supplementary Fig. 1A.

Supplementary Data 2: List of differentially expressed genes of CD4+ T cells, CD8+ T cells, Innate T cells, and B cells. Differential gene expression analysis was performed using the Wilcoxon Rank Sum test.

Supplementary Data 3: List of gene signatures from published data used in analyses for CD4+ T cells, CD8+ T cells, Innate T cells, and B cells. The source data and citations are provided in the Data availability statement and Reference section of the manuscript.

Supplementary Data 4: Table summary of flow cytometry and CITE-seq antibodies used in this study.

Supplementary Data 5: Human Leukocyte Antigen (HLA) information of each patient.
